# RETRACTION: A Randomized, Participant‐ and Evaluator‐Blinded, Matched‐Pair, Prospective Study Comparing the Safety and Efficacy Between Polycaprolactone and Polynucleotide Fillers in the Correction of Crow's Feet

**DOI:** 10.1111/jocd.70794

**Published:** 2026-03-16

## Abstract

**RETRACTION**: ChoiS. Y. , KohY. G. , YooK. H. , HanH. S. , SeokJ. , and KimB. J. , “A Randomized, Participant‐ and Evaluator‐Blinded, Matched‐Pair, Prospective Study Comparing the Safety and Efficacy Between Polycaprolactone and Polynucleotide Fillers in the Correction of Crow's Feet,” Journal of Cosmetic Dermatology 24, no. 1 (2025): e16576, 10.1111/jocd.16576.39313949 PMC11754925

The above article, published online on 23 September 2024 in Wiley Online Library (wileyonlinelibrary.com) has been retracted by agreement between the authors; the journal Editor‐in‐Chief, Michael H Gold; and Wiley Periodicals, LLC. The retraction has been agreed following the authors' request. The authors informed the journal that a misidentification occurred regarding the dermal filler used in the clinical study. Specifically, the product DLMRO1 dermal filler was misidentified as containing pegylated polycaprolactone (PCL), whereas it is composed solely of pure PCL filler. Because pure PCL and pegylated PCL are distinct materials with different biocompatibility, viscosity, and biodegradation profiles, the misidentification significantly affects the reported methods and undermines the reliability of the study's findings. Accordingly, the article has been retracted.

**RETRACTION**: S. Y. Choi , Y. G. Koh , K. H. Yoo , H. S. Han , J. Seok , and B. J. Kim , “A Randomized, Participant‐ and Evaluator‐Blinded, Matched‐Pair, Prospective Study Comparing the Safety and Efficacy Between Polycaprolactone and Polynucleotide Fillers in the Correction of Crow's Feet,” Journal of Cosmetic Dermatology 24, no. 1 (2025): e16576, 10.1111/jocd.16576.39313949 PMC11754925

The above article, published online on 23 September 2024 in Wiley Online Library (wileyonlinelibrary.com) has been retracted by agreement between the authors; the journal Editor‐in‐Chief, Michael H Gold; and Wiley Periodicals, LLC. The retraction has been agreed following the authors' request. The authors informed the journal that a misidentification occurred regarding the dermal filler used in the clinical study. Specifically, the product DLMRO1 dermal filler was misidentified as containing pegylated polycaprolactone (PCL), whereas it is composed solely of pure PCL filler. Because pure PCL and pegylated PCL are distinct materials with different biocompatibility, viscosity, and biodegradation profiles, the misidentification significantly affects the reported methods and undermines the reliability of the study's findings. Accordingly, the article has been retracted.

